# Visual feedback of hand and target location does not explain the tendency for straight adapted reaches

**DOI:** 10.1371/journal.pone.0206116

**Published:** 2018-10-24

**Authors:** Fatemeh Zahed, Max Berniker

**Affiliations:** Department of Mechanical and Industrial Engineering, University of Illinois at Chicago, Chicago, IL, United States of America; University of Exeter, UNITED KINGDOM

## Abstract

Subjects in laboratory settings exhibit straight hand paths—typified by the minimum jerk path—even in the presence of a learned but disturbing force field. At the same time it is known that in this setting, visual feedback strongly influences reaches, biasing them to be straight. Here we examine whether or not this bias can account for the straightness of movements made in a force field. We ran three curl field experiments to investigate how the lack of visual feedback influences adapted reaches. In a first experiment, hand position was displayed at the beginning and at the end of each trial, but extinguished during movement, and the hand was passively brought back to the home location. In the second experiment, visual feedback of neither the hand nor the target was provided, and targets were haptically rendered as “dimples.” In order to provide extended practice, a third experiment was run with a single target and an active reach back to the home location. In all three cases we found minor changes in the adapted reaches relative to control groups that had full visual feedback. Our subjects adopted trajectories that were better explained by minimum jerk paths over those that minimize effort. The results indicate that for point-to-point reaching movements the visual feedback, or lack there of, cannot explain why reaches appear to be straight, even after adapting to a perturbing force field.

## Introduction

Examining how point-to-point reaching movements are controlled and learned is crucial to understanding the motor system. In particular, the kinematics of a movement can reveal important information about how reaches are controlled [1, 2]. One consistent finding is that in reaching movements subjects move their hands along straight paths well approximated by a minimum jerk trajectory [3, 4]. This is true under a wide range of circumstances [2, 5–8]. Even when combating large forces subjects exhibit a strong preference to move like their straight, unperturbed reaches [9–15]. Indeed, even congenitally blind subjects make straight point-to-point reaches in the laboratory setting [16, 17]. Aside from an interesting behavioral phenomenon, these findings suggest the motor system may plan and control movements in terms of the hand’s kinematics, and be largely insensitive to the required kinetics.

While describing the many experimental findings is straightforward, explaining their origins has proven difficult. Our joints do not translate, but rather rotate, at least naively implying curved reaches ought to be the norm. When examining reaches in the context of efficiency, straight reaches are costly for many definitions of mechanical effort. For example, reaches that minimize the rate of joint torques [18, 19], or motor commands [20], or energy [21] or metabolic costs [22], all rely on hand paths with varying degrees of curvature. For short, unperturbed reaches however, these hypotheses predict relatively straight movements. As such, examining perturbed movements, like those made in a force field, are all more important for understanding motor control. Thus, while there is ample evidence that human subjects typically make straight reaches, even under perturbed conditions, there are good reasons to believe that curved ones ought to be the norm.

For practical and scientific reasons studies typically employ a restrictive visual feedback paradigm, limiting vision of the limb and asking subjects to move a cursor on a plain display. It is known that under these conditions visual feedback has a strong influence on the reaches made. For example, subjects will move their hand along a curved trajectory if the resulting visual feedback takes a straight path [5, 23]. In addition, while some studies with reduced visual feedback find that adapted reaches are relatively straight [10, 12], there is also evidence that reduced visual feedback results in curved adapted reaches [24]. It is well established that visual feedback influences reaches, what is not is clear is if this influence can account for the lack of curvature observed after adapting to perturbations. Importantly, while the effects of reduced visual feedback on adapted reaches have been explored, reaches made in the absence of visual feedback have not.

We designed three force field adaptation experiments to examine whether reducing visual feedback results in adapted hand paths distinct from those made with the standard feedback. First we ran an experiment to see how adapted hand paths differed with and without visual feedback during movement. In the second experiment, we went further and extinguished visual cues of the hand and target entirely. In the third experiment we extended practice by reducing the number of targets to one, and eliminated a passive reach to the home position just in case it introduced a bias for straight movements. A few significant differences in reach curvature were found, but on the whole we could not conclude that adapted reaches without visual feedback were distinct. A subsequent model-based analysis concluded the reaches in each experiment were better described as straight, minimum jerk-like movements rather than curved, efficient movements. Our findings suggest the visual feedback provided cannot explain why reaches are controlled to be straight after adapting to a perturbing force field.

## Methods

### Subjects

Seventy healthy, right-handed adults (mean age, 24, standard deviation, 4.1, 24 females) naïve to the purposes of the experiments participated in the study. Handedness was assessed using the Edinburgh Inventory [25]. Subjects signed consent forms prior to participating and were paid a baseline compensation plus a bonus proportional to their performance (ranging from $10-$25). All experimental protocols were approved by the University of Illinois at Chicago’s Office for the Protection of Research Subjects.

### Experimental apparatus

A robotic manipulandum (BKIN’s KINARM End-Point Lab) was used to generate forces on the subject’s hand and also to record kinematic and force data. Custom-written software for the experiment was run in the real-time xPC platform at 2kHz. On each cycle hand position and velocity were computed using the robot’s joint angles and filtered with a 2^nd^ order Butterworth filter with a 50Hz corner frequency. On each trail a null field or a velocity-dependent curl field (with a strength of 20Ns/m) was used to compute a desired force, F_desired_. A low-gain force-feedback loop was used to help render this desired force, compensating for the inertial dynamics of the manipulandum. The resulting commanded force was,

$$F_{robot}=F_{desired}+K(F_{desired}-F_{measured})$$

where *K* = 0.5 for the null field, 0.75 for the curl field, and F_measured_ is the force transducer measurement. The measured force and commanded force, F_robot_ were also filtered with a 2^nd^ order Butterworth filter with a 50Hz corner frequency. For post processing raw hand position and forces were saved at 1kHz.

### General experimental protocol

**Fig 1:**
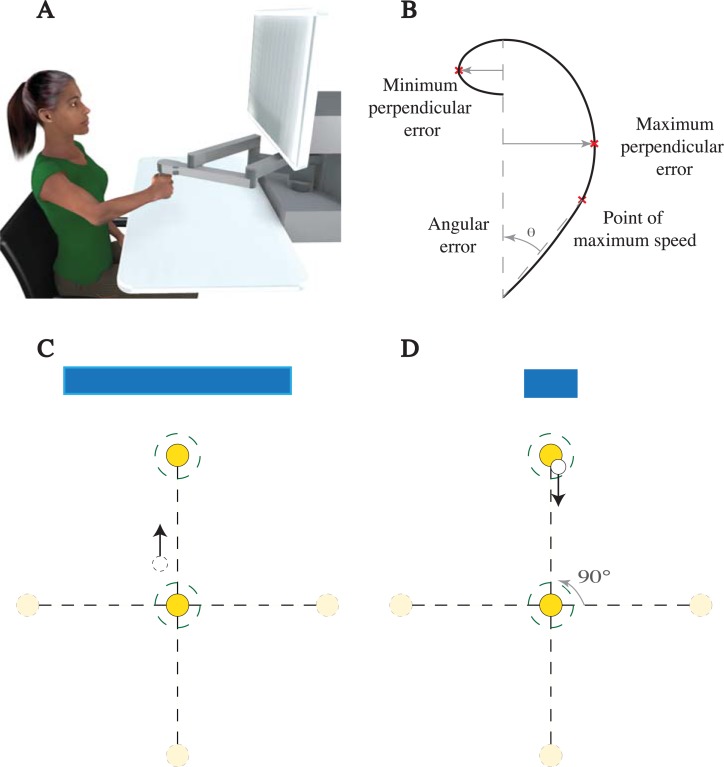
Experimental setup. A) Subjects made point-to-point reaching movements holding the handle of a manipulandum with their dominant, right hand. Their arm was approximately in a plane aligned with their shoulder and supported from above by two slings (not shown). B) A depiction of three reach metrics, maximum perpendicular error, minimum perpendicular error and angular error. C) and D) Reaches were made to one of four targets in a pseudorandom order. In experiment I a white cursor displayed the hand’s location when it was within 7.5mm (dashed circles) of the home or target. The length of an error bar on the top of the screen was proportional to the distance between the hand and the target.

Subjects were seated in a height adjustable chair to comfortably view the screen in front of them and grip the handle of the manipulandum with their dominant right hand. Their arms were in a horizontal plane roughly aligned with their shoulder (Fig 1A), and supported by two slings suspended from the ceiling. On each trial, subjects reached from the home position to a target, came to a complete stop, and then moved back. During these reaches visual feedback of the hand’s cursor was extinguished (see details below). Subjects were told that during some trials they would feel a disturbing force. No specific instructions on how to move were provided, they were merely told to come to a stop within the target and within the correct time.

In Experiments I and II subjects reached to one of four pseudo randomly drawn targets 10cm away (0°, 90°, 270° and 360° directions, see Fig 1C). In Experiment III, subjects reached to one target (90°). A trial began when a target appeared on the screen and ended when their hand landed within the target with a speed less than 0.05m/s. Successful trials ended within a window of 900±100ms. Feedback of performance was provided at the end of each trial, indicating if the movement was too fast, too slow, or correct. In the first two experiments the subjects were passively dragged back to the home position by the robot. In the third experiment subjects actively reached back to the home position. After a random pause (0-200ms) the next trial began. After each trial the subject’s cumulative score was displayed in the top corner of the screen.

Each experiment was broken into three sections: baseline (reaches in the null field), adaptation (reaches in the force field) and washout (reaches in the null field). In the adaptation section two out of every fifteen trials, drawn randomly, were catch trials in which the force field was unexpectedly replaced with the null field. In total each subject performed 920 trials for Experiments I and II and 750 trials for Experiment III (see Table 1). Each experiment had an identical control group with continuous visual feedback of the hand and target.

**Table 1:** Details of the three experimental protocols.

	**Number of targets**	**Number of baseline trials**	**Number of adaptation trials**	**Number of washout trials**	**Reach back**	**Visual feedback of target and cursor**
Experiment I	4	120	600	200	Passive	Partial
Experiment II	4	120	600	200	Passive	No
Experiment III	1	150	450	150	Active	No

### Experiment I

Twenty subjects were randomly divided into two groups of ten. The home and target position were displayed as 10mm diameter yellow circles, while the hand’s location was displayed with a 5mm diameter white cursor (see Fig 1C). In the main group, the cursor would vanish once the hand was 7.5mm away from the home position and would turn on again when within 7.5mm of the target (the visible regions are shown as dashed circles in Fig 1C). To help subjects find the targets while the cursor was not visible, an “error bar,” whose length was proportional to their distance (2.5 times the actual distance) to the target, was displayed on the top of the screen. The length of the error bar changed in real time with hand position (Fig 1D). At the end of a trial, the target and the bar would change color to provide feedback of performance. Green, blue and red colors were used to describe successful, too slow and too fast movements, respectively. A point would be awarded for each successful reach.

Subjects performed 120, 600 and 200 trials in the baseline, adaptation and washout sections, respectively (see Table 1). All subjects rested for approximately 3–5 minutes half way through the adaptation section. The control group was identical, with the exception that the cursor was provided continuously throughout each trial of the experiment.

### Experiment II

**Fig 2:**
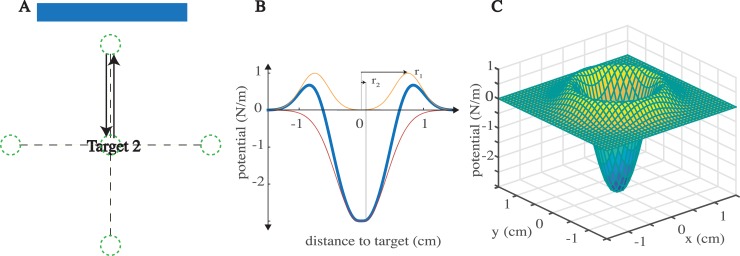
Setup for Experiment II. A) Reaches were made to one of four targets. Neither the targets nor the hand location were displayed. An error bar displayed the distance between the hand and the target. Targets were rendered as virtual dimples. B) A cross section through the dimple's defining potential functions, a "ridge" (orange line) and a "hole" (red line) which when summed create our dimple (blue line). C) A 3D view of the virtual dimple's potential function.

Twenty subjects were randomly divided into two groups of ten. In the main group, neither the cursor nor the targets were shown to the subjects. Instead, a target number was displayed on the screen (numerals 1–4) and targets were rendered as “dimples” (described below). Subjects were informed of the target numbers and the dimples prior to the experiment. As with Experiment I, the error bar was displayed to help guide subjects to the target (Fig 2A). Successful reaches were defined similarly, however feedback on reach performance was delivered via color-coded changes in the error bar. The control group had identical dimpled targets and continuous visual feedback.

To provide an alternative to the visual feedback, we used haptic feedback of the home and target positions. Each target was a small virtual “dimple,” rendered with forces applied to the hand. As the hand neared a target, subjects would feel a circular “ridge” around a small “hole” (Fig 2B). We defined the dimple using a potential energy function, *U*, describing its energy in terms of the distance from the target center (Fig 2C). The potential function included one term for the outer ridge, and another term for the central hole. Expressed in radial coordinates it is,

$$U(r)=\exp\left(-10{\left(r-r_{1}\right)}^{2}\right)-3\;\exp\left(-4{\left(r-r_{2}\right)}^{2}\right)$$

where *r = [x*^*2*^*+y*^*2*^*]*^*1/2*^ is the hand’s distance from the center of the target, *r*_*1*_ is the radius of the outer ridge, 0.75cm, *r*_*2*_ is the radius of the central hole, 0.05cm. *U*(*r*) is set to a constant value when *r* < *r*_*2*_, creating a dead-zone (Fig 2B). The force exerted on the hand was the negative gradient of this potential function.

$$[f_{x},f_{y}]=-\left[\frac{\partial U}{\partial x},\frac{\partial U}{\partial y}\right]$$

Subjects had the same schedule of trials as in Experiment I (see Table 1). All subjects rested for approximately 3–5 minutes half way through the adaptation section. In the control group the cursor and target locations were displayed continuously throughout the trials.

### Experiment III

Thirty subjects were randomly divided into two groups of fifteen. In this experiment subjects only reached to the 90° target. In the main group, neither the cursor nor the target was shown to the subjects. Instead, the same error bar as in previous experiments was provided, and the home and target positions were rendered with dimples. Unlike Experiments I and II, the robot did not pull the subject’s hand back to the home position after a reach. Instead, subjects actively reached back to the home position after the performance feedback (same color-coded changes in error bar as the previous experiment) was provided.

Subjects performed 150, 450 and 150 trials in the baseline, adaptation and washout sections, respectively (see Table 1). Subjects rested for approximately 3–5 minutes every 150 trials. The first 10 trials of the adaptation section (trials 151–160) were null trials, to observe the transition from null to force field trials after the rest break, and were not considered in our analysis. Similarly, the first 10 trials of the washout section were force field trials and were not included in our analysis. The control group was identical (with dimples), except the cursor and target location were displayed continuously throughout the trials.

### Data analysis

We computed five reach metrics for each trial. The first three metrics quantified how much reaches deviated from a straight line connecting the home and target positions (Fig 1B). The maximum and minimum perpendicular deviations from this line were computed, to measure the deviations in the direction, and in the opposite direction, of the perturbation (abbreviated max-perp, and min-perp, respectively). We do this to distinguish between deviations due to the field, and those due to reach overshoot in the opposite direction. We define perpendicular error in the direction of the force field as positive. It should be noted that the minimum perpendicular errors are not necessarily negative (e.g. if the reach’s path is entirely in the direction of the filed), however, in all cases the averages were negative.

Angular error (abbreviated ang-error) was the angle between the straight line to the target, and a line from the home position to the point on the hand’s path of maximum speed (see Fig 1B). We also computed the maximum speed and force.

These five metrics were computed using hand and force data collected at 1KHz. Velocity was computed with a back-difference approximation of hand position and smoothed with a 5^th^ order Butterworth filter with a corner frequency of 20Hz. All metrics were analyzed independent of reach direction.

Statistical comparisons were examined on averages computed over 5 blocks of data. Each block consisted of 40 consecutive trials measured in late baseline (the last 40 trials), early adaptation (the first 40 trials), late adaptation (the last 40 trials), early washout (the first 40 trials) and late washout (the last 40 trials of the experiment). Catch trials were excluded from early adaptation and late adaptation. Tests for significance were performed across blocks using paired t-tests and ANOVA’s. The significance level was set to 0.05, but Bonferroni corrected for 3 comparisons (*p* = 0.016) for the t-tests, and 5 comparisons (*p* = 0.01) for the ANOVA tests.

To operationally define curved reaches we utilize a model-based prediction. We model the limb as a two-link arm with fixed shoulder position, subjected to a clock-wise curl-field. The equations of motion are:

$$T+{J(\theta )}^{T}F=I(\theta )\ddot{\theta }+C(\theta ,\dot{\theta })\dot{\theta }$$

where *θ = [θ*_*s*_, *θ*_*e*_*]*^*T*^ is the shoulder and elbow angel, *T* is the commanded torque to the shoulder and elbow, *F* is the curl field force, *J* is the Jacobian matrix. *I* and *C* are the standard matrices for the limb inertia and the Coriolis and centripetal acceleration terms[26]. The limb parameters, link lengths, mass, distance to center of mass and moment of inertia, were set to (0.31, 0.3)*m*, (2.1, 1.65)*kg*, (0.152, 0.2465)*m* and (0.0264, 0.0472)*kg-m*^*2*^ for the upper and lower arm, respectively [27].The curl field is defined by the hand’s velocity

$$F_{CF}=\alpha \;\left[\begin{array}{cc} 0 & 1\\ -1 & 0 \end{array}\right]\;[v_{x},v_{y}]$$

where α is set to 20*Ns/m*. We simulated the same 10*cm* reach made by our subjects with a similar average movement time of 0.8 seconds. With the shoulder at the origin, the home position was at (-20, 20)c*m*, approximately the same location as the subjects.

**Fig 3:**
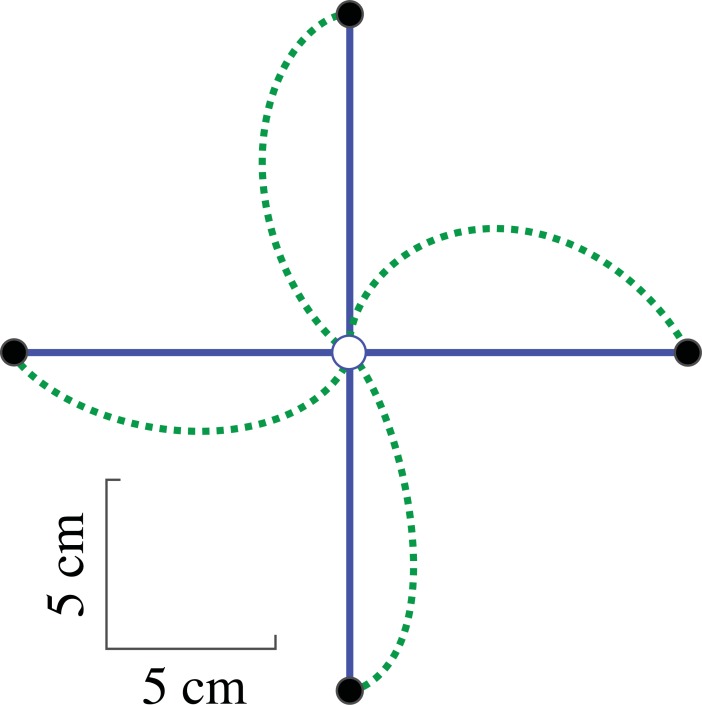
Model predictions. Center-out reaches are displayed for four targets (black circles). Each of the paths represents the optimal solution for a different cost function. The solid blue line is the minimum jerk path and the dotted green line is the path for minimum rate of commanded torques. The home position (white circle) is in the (-20,20) *cm* location with respect to the shoulder/origin (not shown).

A minimum jerk movement was used as a reference for straight reaches. A movement that minimized the rate of joint torque,

$$C=\frac{1}{2}\int_{0}^{t_{f}}{\left[(d\tau_{e}/dt\right)}^{2}+{\left(d\tau_{s}/dt\right)}^{2}]dt$$

was used as a stand-in for mechanically efficient reaches, and a reference for curvature (see Fig 3).

To categorize movements as being better described as either straight, or curved, we compute the likelihood of each model given our experimental data. To do so we assume the data is normally distributed, with a mean and covariance given by the across subject average trajectories and their standard error of mean’s (SEM). For each model we compute the likelihood of the predicted trajectory. This is found by computing product of the likelihood of each data point at 0.07-second intervals. A subsequent likelihood ratio test was used to quantify a model’s relative goodness of fit using a chi-squared (χ^2^) distribution and Wilks’ theorem. The *p*-value determines whether the minimum rate of torque model is significantly a better fit to the data than the minimum jerk model. This test determined if the data was better described as “straight” or “curved,” in the context of our model for efficiency.

## Results

To examine if the lack of visual feedback alters adapted point-to-point reaching movements, we conducted three experiments. In the first experiment visual feedback of the cursor was provided only in the beginning and end of the movement, while in the second experiment no visual feedback of the cursor or the target was provided at all. To determine if further practice would alter the results, a third experiment, similar to the second but with a single target was conducted. Each experiment had a control group with continuous visual feedback. To quantify differences across experimental conditions we compared three reach metrics on each trial: the maximum and minimum perpendicular deviations and the angular deviation from a straight line. The maximum and minimum perpendicular errors allowed us to make a clear distinction between deviations in the direction of the force field, and those in in the opposite direction (due to overshoot or movements in a catch trial). For comparisons across experiments and groups we also computed the peak speed and the peak force of each trial.

Our overall behavioral findings are similar to what is commonly observed in force field adaptation. In all three experiments, reaches in baseline were relatively straight. Early reaches in the force field trials were curved, revealing the effects of the disturbing field, and with practice became less so. In washout, reaches were curved in the opposite direction, and with practice became straight once again. In general subjects exhibited typical force field adaptation behavior despite the lack of typical feedback cues.

### Experiment I

**Fig 4:**
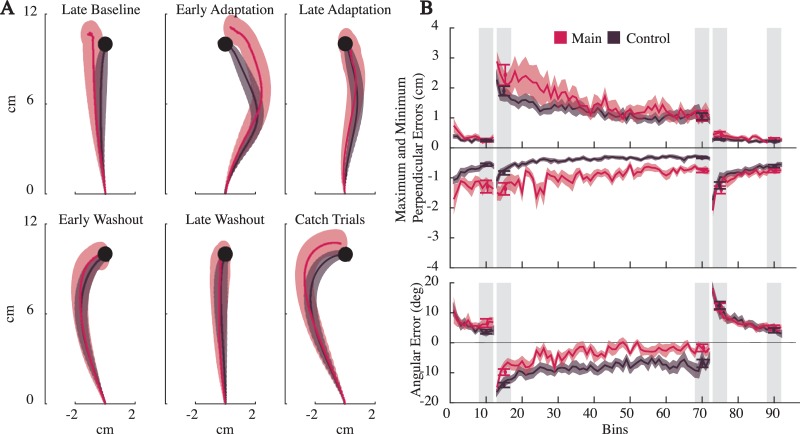
Results for Experiment I. Pink and gray colors represent the main group and control groups, respectively. A) The across-subject average trajectories (mean ± SEM) for each block. Note that since the trajectories are averaged across time, a varying time horizon was used for each block to ensure a minimum amount of data from all subjects was available. As a result some average trajectories end before reaching the target. B) The across-subject reach metrics (mean ± SEM), in 10 trial bins. Also displayed, are the averages for the blocks (mean ±SEM), obtained using the 40 trials indicated with light grey vertical strips. All metrics were subtracted by their mean baseline value for statistical comparisons.

As is clear from the average trajectories (Fig 4A), subjects experiencing the force field initially made curved reaches, which progressively became relatively straight. Early washout trials were curved in the opposite direction of the field and become straight again by the end of the experiment. When comparing the two groups, the perpendicular errors in the control group (with full visual feedback), were consistently smaller than the main group (Fig 4B). The angular errors, however, were larger for the control group. To examine this we compared the points along the path where angular error was computed (the point of peak speed). We found that the time of peak speed for the control group was earlier in the reach than the main group (0.22 seconds for the control, and 0.25 seconds for the main), potentially playing a part in these differences.

To verify that subjects adapted to the force field in each group, we compared the reach metrics from the late baseline block with those from the second half of the catch trials. In both our main group and the control group, the minimum perpendicular error (in the opposite direction of the field) and angular error were significantly larger in catch trials (paired *t*-tests for min-perp error and ang-error were, *p* = 3.1E-5, 8.1E-8 for the main group, and *p* = 5.7E-9, 3.6E-9 for the control group), suggesting subjects were expecting the force field. The maximum perpendicular errors of baseline and the catch trials were not significantly different (*p* = 4.9E-2 for the main, and *p* = 8.9E-2 for the control). This is not surprising since the maximum perpendicular error measured deviations in the direction of the field, which was absent in both cases. Thus our analysis of catch trials, which curved in the opposite direction of the force field, are consistent with adapted movements, even with limited visual feedback.

To further verify that subjects adapted to the force field, we compared reaches during early adaptation and late adaptation (see Fig 4A and 4B). All reach metrics significantly decreased across these two blocks for the main group (*p* = 2.3E-3, 9.7E-3, 8.1E-5 for max-perp error, min-perp error and ang-error respectively). This was also true for the control group (*p* = 3.6E-4, 1.0E-6, 1.7E-4). These results further demonstrate that with limited visual feedback subjects adapt to a perturbing force field.

Next we used our model predictions to determine if the averaged trajectories during the late adaptation block were better described as straight, minimum jerk-like movements or curved, efficient movements. A likelihood ratio test revealed the minimum jerk movement was a superior description of the data (*p ≈* 0) for both the main and control group.

The above analyses make it clear that the subjects with reduced visual feedback adapted to the force field, but did they adapt differently than those with visual feedback? To answer this, we ran a two-way ANOVA comparing the effect of feedback type, and blocks (to control for the changes throughout the experiment), on the reach metrics. To compare differences in groups relative to their nominal performance, the late baseline values were subtracted from all metrics. For this final comparison, we also included peak speed and peak force. The results found little effect of visual feedback on reaches. No significant difference was found for any of the metrics except the minimum perpendicular error, which measured deviations in the opposite direction of the field (F_(4,1)_ = 3.08, 0.21, 0.1, 0.78, *p*>0.01 for max-perp error, ang-error, peak speed and peak force, F_(4,1)_ = 13.38, *p* = 4E-4 for min-perp error). The results indicate that visual feedback had little influence on reaches.

To determine if the main and control group exhibited the same degree of adaptation, we compared their late adaptation metrics relative to their late baseline values. In addition to the min-perp, max-per and ang-error, we also compared the mean peak speeds (0.32 +/- 0.02 *m/s* and 0.29 +/- 0.01 *m/s* for the main and control group respectively) and mean peak force values (6.39 +/- 0.37 and 5.77 +/- 0.22). Paired *t*-tests found no significant differences for any of the metrics (*p*>0.01). Thus, visual feedback of the hand and target did not have an affect on the degree of adaptation.

On the whole, while small quantitative differences between the main and control group were found, the average trajectories of late adaptation were qualitatively similar in both groups (see Fig 4A). Only one of the 10 statistical tests examined found a significant difference between groups. If we had limited our comparisons to the typical reach metric, an unsigned perpendicular error, subjects with and without visual feedback would be indistinguishable. Thus we conclude that, as found in prior studies, with limited visual feedback subjects are able to adapt to a perturbing force field [24, 28, 29], and do so by returning to relatively straight reaches.

### Experiment II

In the previous experiment we saw that by partially removing feedback, small but distinct differences could be found in the reaching patterns. This raised the possibility that removing more of the visual feedback might reveal even larger differences. To examine this possibility we ran a new experiment completely lacking feedback of the cursor and target. Instead, subjects used an “error bar” and haptically rendered targets (see Methods). We performed the same comparisons here as was done in Experiment I, examining perturbed reaches without visual feedback against a control group with continuous visual feedback.

**Fig 5:**
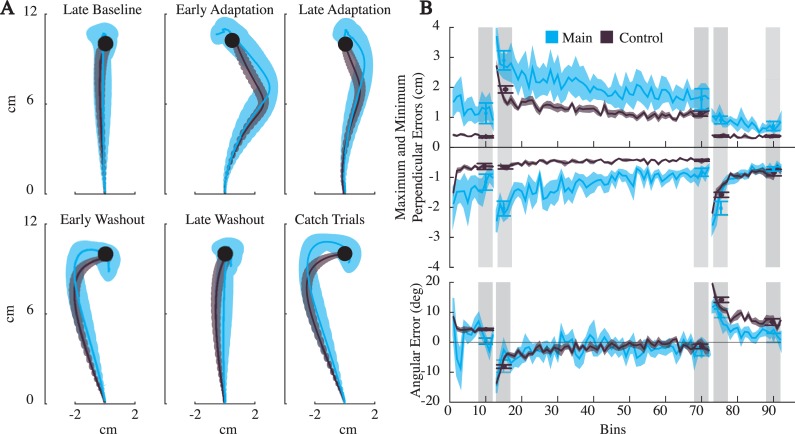
Results for Experiment II. Blue and gray colors represent the main group and control groups, respectively. A) The across-subject average trajectories (mean *±* SEM) for each block. Note that since the trajectories are averaged across time, a varying time horizon was used for each block to ensure a minimum amount of data from all subjects was available. As a result some average trajectories end before reaching the target. B) The across-subject reach metrics (mean *±* SEM), in 10 trial bins. Also displayed, are the averages for the blocks (mean *±*SEM), obtained using the 40 trials indicated with light grey vertical strips. All metrics were subtracted by their mean baseline value for statistical comparisons.

As is clear from the average trajectories (Fig 5A), reaches were qualitatively similar to Experiment I. We compared the reach metrics from the baseline block with the second half of catch trials. Minimum perpendicular errors and angular errors were significantly different across blocks for the main group (*p* = 3.2E-6, 2.4E-6) and the control group (*p* = 8.4E-12, 2.1E-13). As with Experiment I, the maximum perpendicular errors, measuring deviations in the direction of the absent field, were not different across blocks for either group (*p* = 8E-1 and *p* = 6.3E-1 for the control). Thus, as is typical after adapting, subjects in both groups were expecting the force field.

Next we compared early and late adaptation blocks (Fig 5A and 5B) for further indications of adaptation. Maximum and minimum perpendicular errors significantly decreased across the course of adaptation (*p* = 1.4E-2, 1.5E-4 respectively, and *p* = 3.1E-5, 2.5E-3 for the control group). Angular error also decreased, however the effect was not significant for the main group (*p* = 7.0E-2, and *p* = 1.4E-6). These results suggest that even in the complete absence of visual feedback of the hand and target, subjects can adapt to a perturbing field.

We computed a likelihood ratio test for our two models and again found the straight reaches of a minimum jerk model better describing the data for both groups (*p ≈* 0). Here again, we conclude these reaches were relatively straight when viewed in the context of efficiency.

As with Experiment I, we made a direct comparison between the main group and its control to test the influence of visual feedback. Here again we included all 5 metrics, and compared them relative to their mean late baseline values. A two-way ANOVA revealed that the lack of feedback only had a significant affect on peak force (F_(1,4)_ = 1.9, 0.06, 1.87, 6,91 all *p*>0.01 for max-perp error, min-perp error, ang-error and peak speed, and F_(1,4)_ = 15.74, *p* = 1E-4 for peak force). Measurements revealed that by the end of the adaptation block peak forces were larger in the main group (9.0N ± 0.5 and 6.5N ± 0.06). Thus when visual feedback of both the target and cursor is absent, reaches (throughout the experiment) are similar to their standard, full visual feedback counterparts.

Our final comparison was of the amount of adaptation for the main and control groups. The mean peak speed and peak forces values (not shown) were 0.47+/-0.03 *m/s* and 0.35+/-0.02 *m/s*, and 9.0+/-0.5 *N* and 6.5+/-0.06 *N* for the main and control group respectively. Relative to the baseline values, the peak force in late adaptation was significantly different between the two groups. However, none of the remaining four metrics were significantly different (*p*>0.01). On the whole, we conclude that subjects in the two groups adapted by the same amount.

As with the previous experiment we did find small quantitative differences between the main and the control group (two out of the 10 statistical tests were significant). Overall we cannot conclude that the two groups are distinct, and suggest that in the absence of visual cues of the hand and the target, adapted reaches are similar to those with continuous visual feedback and relatively straight.

### Experiment III

Experiment III addressed two potential concerns that may have influenced adapted reach paths. First, it could be the case that with extended practice subjects become more familiar with the force field, and alter their reaches relative to the control group. To address this we used a single target to provide more focused practice. Second, it could be the case that passively dragging the subject's hand to the home position along a straight path influenced their behavior, implicitly cueing them to follow a similarly straight path outwards. Therefore, in this experiment we had subjects actively reach to the home position after each trial. As with experiments I and II, an identical control group was run with continuous visual feedback for comparison.

**Fig 6:**
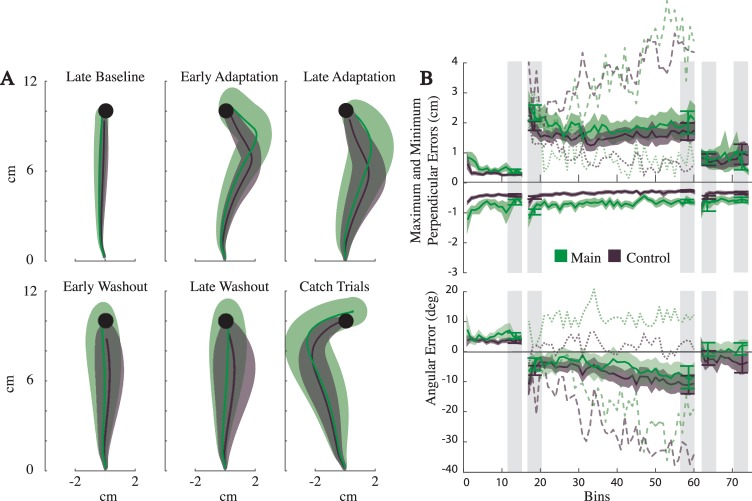
Results for Experiment III. Green and gray colors represent the main and control groups, respectively. A) The across-subject average trajectories (mean *±* SEM) for each block. Note that since the trajectories are averaged across time, a varying time horizon was used for each block to ensure a minimum amount of data from all subjects was available. As a result some average trajectories end before reaching the target. B) The across-subject reach metrics (mean *±* SEM), in 10 trial bins. Also displayed, are the averages for the blocks (mean *±*SEM) obtained using the 40 trials indicated with light grey vertical strips. Dashed lines represent the means for four example subjects (see Fig 7). All metrics were subtracted by their mean baseline value for statistical comparisons.

The average trajectories in this experiment followed the same general trend as in the previous two experiments (Fig 6A), with the notable exception of larger variability (see below). To verify that the subjects adapted, we compared the late baseline block with the second half of the catch trials. In the main group, maximum and minimum perpendicular errors were significantly different while the angular error was not (*p* = 1.1E-3, 1.6E-5 and 5.5E-1 respectively). In the control group, minimum perpendicular error and angular error were significantly different and the maximum perpendicular error was not (p = 4.1E-7, 3.0E-3 and 2.2E-1 respectively). Relative to Experiments I and II, these findings were more mixed, but suggestive of the findings commonly observed after adaptation.

We further tested adaptation by comparing reaches in the early and late adaptation blocks (Fig 6A and 6B). The minimum perpendicular error was significantly different for both groups (*p* = 2.5E-3 and *p* = 5.5E-4 for the control). However, the maximum perpendicular error and angular error, measuring the reach curvature due to the field, were not significantly different (*p* = 4.8E-1, 3.1E-1, and *p* = 5.6E-1, 1.7E-1 for the control). Therefore, despite the extended practice, and in contrast with the prior two experiments, we did not find clear evidence for decreased errors by the end of the adaptation block.

**Fig 7:**
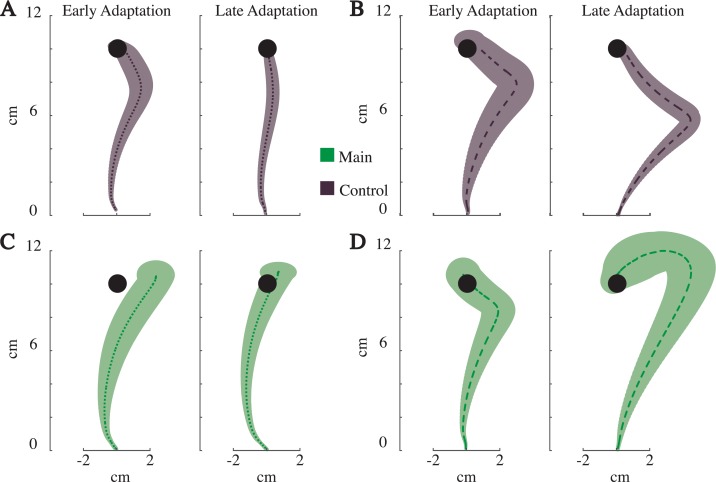
Example subject’s results for Experiment III. A), B) Average trajectories (mean*±* SEM) for early and late adaptation blocks for two example subjects from the control group. C), D) Average trajectories (mean*±* SEM) for early adaptation and late adaptation blocks for two example subjects from the main group.

As mentioned above, the variability across subject trajectories was relatively large, and their paths were inconsistent in both the main and control groups. To illustrate, we present the average trajectories for four illustrative subjects (Figs 6B and 7A–7D). As is standard, two of the four subjects made progressively smaller errors during the adaptation block and became straighter (see Fig 6B dotted lines, and Fig 7A and 7C). The other two, however, made progressively larger errors, curving in the direction of the field (see Fig 6B dashed lines, and Fig 7B and 7D). It is interesting to note how one subject curves into the field (Fig 7C), as would be predicted by an effort to minimize the forces required to move and accelerate the limb, whereas the other subject appears to yield to the field forces (Fig 7D). Regardless of the interpretation, these inconsistencies made it difficult to examine the across–subject averages for clear signs of adaptation.

To explore this variability in reaching paths, we asked whether an individual subjects’ adapted trajectories were related to their handedness, as quantified by the Laterality Quotient (L.Q.) of the Edinburgh’s Handedness Test. However, no significant correlations were found with any of the reach metrics during late adaptation, in either group (all *p*>0.01).

As with the previous experiments, we used our model predictions to test whether the minimum jerk model or the minimum rate of commanded torques better predicted adapted reaches. The results of the likelihood ratio test (*p ≈* 0) again found that reaches were better described as straight, minimum jerk-like movements rather than the curved movements predicted for efficient reaches.

Once again we compared the main group and its control to determine if the lack of visual feedback influenced reaches. As before, all 5 metrics were examined relative to their baseline values. An ANOVA found no significant effect on any of the metrics (*p*>0.01). Reaches were quantitatively similar whether visual feedback was provided or not.

Finally we compared both groups’ late adaptation metrics relative to their baseline values to determine if there were differences in the amount of adaptation. For this test we included the peak speeds (mean+/- standard error, 0.4+/-0.02 *m/s* and 0.35+/-0.01 *m/s*) and peak forces (8.2+/-0.44 *N* and 7.4+/-0.564 *N*) for the main and control group respectively. Paired *t*-tests found no significant differences for any of the metrics (*p*>0.01), demonstrating that the subjects in both groups adapted to the same degree. Thus we conclude once more, that the absence of visual feedback of the hand and target did not have an affect on adapted reaches.

## Discussion

Here we examined the effects of visual feedback on adapted reaches in a force field. In Experiment I we found reaches were similar with and without continuous visual feedback. Only one of ten tests (a change in the minimum perpendicular error) resulted in a significant difference between groups. The results from Experiment II, where the main group had no cursor feedback, were similar and only the peak forces were significantly different. In Experiment III, with extended practice on a single target, subjects’ reaches were more variable in both the main and control group; some subjects curved away from the field, heading in the CW direction relative to the target, while others headed in the CCW direction. Yet, on average no significant differences were found between the two groups. Importantly, a number of reach metrics indicate that these reduced feedback conditions did not hinder learning. Thus the results cannot be explained as a consequence of incomplete adaptation. We concluded that on the whole, and on average, adapted reaches, with or without visual feedback of hand and target location are straight and better explained by a minimum-jerk model rather than a model that minimizes the effort of the reach.

In general, with or without the presence of external forces, a reach that minimizes the effort of movement (e.g. minimizing commanded torques, or torque rates, or energy, or metabolic cost) should result in varying degrees of curvature in the hand's path [18, 19, 21, 22, 30, 31]. This is also true for reaches in a velocity-dependent curl field [32]. Yet subjects exhibit a strong preference for straight reaches, despite the presence of large disturbing forces [9–14]. Indeed, even after practicing a curved path that minimizes the energy of a reach in a nonlinear force field, subjects revert to straight reaches when given the chance [10, 15]. Interestingly, a few recent studies have found that reaches are no longer straight when the visual feedback is manipulated [21, 24]. These findings motivated us to investigate the effects of removing any visual cues for the hand and target, perhaps eliminating the preference for, “seeing straight reaches.” With a few notable exceptions in Experiment III, we find that on average after removing visual cues subjects disregard effort and make straight reaches such as those predicted by a minimum-jerk model.

Our experiments did not completely eliminate visual cues. Although it is not obvious if or how it could influence reaches, the error bar was visually displayed. As with a displayed cursor, a straight reach to the target would be the quickest way to reduce the size of the error bar, thus potentially biasing the movements. A possible solution for eliminating this last visual cue is to replace the error bar with another feedback signal such as an auditory tone, a larger dimple or similar haptic cue. Future work can investigate these possibilities to look for changes in adapted reach patterns.

A potential concern is that adapting to a novel force field may be more difficult when visual feedback is absent, and thus impact the results. However, we found that error curves plateaued well before the end of adaptation, and that they converged to values close to that of baseline (with the notable exception of Experiment III, where there was large variability across subjects). Prior studies have also shown that with reduced visual feedback, subjects are still able to adapt to a force field [28, 29]. While the absence of visual feedback does not appear to be a barrier for adaptation, future experiments can investigate the effects of extended practice without feedback, on path curvature.

A notable finding was the differences in adapted reaches observed in Experiment III. Unlike Experiment II which also had no visual cues for the hand and target, here subject’s adapted reaches were qualitatively distinct, some pointing CW and some CCW with respect to the starting target. A possible explanation is that individual subjects display individual preferences for their movements after visual feedback is removed. Perhaps with less information to guide their movements and the extended practice, individual preferences that would normally be masked by experimental conditions are exposed. We hope to examine this possibility in future work.

Setting aside the issue of whether or not visual feedback influences movements, it is worth noting the apparent distinction of velocity-dependent curl fields relative to other experimental perturbations. Movements made under very similar experimental conditions with other dynamical loads, such as masses and springs, do indeed arc the hand in an energetically efficient manner [21, 30, 33, 34]. This begs the question, why are reaches in the curl field uniquely “non-curved” and inefficient? One can speculate that adapting to the curl field is insufficient to exhibit the kind of efficiency seen with other more familiar loads. Adaptation is likely the first step in the process of acquiring a new motor skill [35], and may not entail the formation of an internal model necessary for efficient movements. If this were the case, then a careful reevaluation of this widespread reaching paradigm and its interpretations would be in order.

This study was motivated by a desire to understand how the central nervous system plans and controls reaching movements. By limiting visual feedback, we hoped to recover what could be natural reaching behaviors in the absence of possible visual biases. Our results reveal that visual feedback of the hand and target cannot explain the strong preference for making straight reaches after adapting to a perturbing curl field.
